# Multimodal porogen platforms for calcium phosphate cement degradation

**DOI:** 10.1002/jbm.a.36686

**Published:** 2019-04-09

**Authors:** Irene Lodoso‐Torrecilla, Eline‐Claire Grosfeld, Abe Marra, Brandon T Smith, Antonios G Mikos, Dietmar JO Ulrich, John A Jansen, Jeroen JJP van den Beucken

**Affiliations:** ^1^ Present address: Department of Regenerative Biomaterials Radboud Institute for Molecular Life Sciences, Radboud University Medical Center 6500 HB Nijmegen The Netherlands; ^2^ Department of Bioengineering Rice University Houston Texas 77030; ^3^ Department of Chemical and Biomolecular Engineering Rice University Houston Texas 77005; ^4^ Department of Plastic & Reconstructive Surgery Radboud University Medical Center 6500 HB Nijmegen The Netherlands

**Keywords:** calcium phosphate cement, degradation, PLGA, porosity, sucrose

## Abstract

Calcium phosphate cements (CPCs) represent excellent bone substitute materials due to their biocompatibility and injectability. However, their poor degradability and lack of macroporosity limits bone regeneration. The addition of poly(d,l‐lactic‐co‐glycolic acid) (PLGA) particles improves macroporosity and therefore late stage material degradation. CPC degradation and hence, bone formation at an early stage remains challenging, due to the delayed onset of PLGA degradation (i.e., after 2–3 weeks). Consequently, we here explored multimodal porogen platforms based on sucrose porogens (for early pore formation) and PLGA porogens (for late pore formation) to enhance CPC degradation and analyzed mechanical properties, dynamic in vitro degradation and in vivo performance in a rat femoral bone defect model. Porogen addition to CPC showed to decrease compressive strength of all CPC formulations; transition of the crystal phase upon in vitro incubation increased compressive strength. Although dynamic in vitro degradation showed rapid sucrose dissolution within 1 week, no additional effects on CPC degradation or bone formation were observed upon in vivo implantation. © 2019 The Authors. *journal Of Biomedical Materials Research Part A* Published By Wiley Periodicals, Inc. J Biomed Mater Res Part A: 107A: 1713–1722, 2019.

## INTRODUCTION

Calcium phosphate (CaP)‐based bone substitutes are the most widely used synthetic alternatives for autologous bone grafts in the field of bone regenerative surgery.^1^ Clinically, CaP‐based bone substitutes have advantages over autologous bone in terms of off‐the‐shelf availability, for which no restrictions in quantity apply and no extra surgical site to harvest donor bone is required.

A cement form renders these CaP‐based bone substitutes injectable, allowing such CaP cements (CPCs) to be applied via minimally invasive surgery and to be molded into the bone defect for optimal bone defect filling.^2^, ^3^ Depending on the end product of the setting reaction, CPCs can be roughly divided in two categories; apatite or brushite. Of those, most research has focused on apatitic CPCs due a higher similarity to the mineral phase of bone and their superior mechanical properties than brushite CPCs.^4^, ^5^ Due to their similarity to the mineral phase of bone, apatitic CPCs possess several advantageous biological properties, including biocompatibility, bioactivity, and osteoconductivity.^6^ However, CPCs have demonstrated to degrade at a slow rate, limiting the available space for the formation of new bone; and hence the complete regeneration of a bone defect.^7^, ^8^ To overcome this drawback, multiple types of porogens have been explored as an additive for CPC,^8^ of which poly(lactic‐co‐glycolic‐acid) (PLGA) has demonstrated to be most effective.^9^ PLGA porogens have a beneficial (hydrolytic) degradation profile, which generates porosity within the ceramic CPC matrix and simultaneously accelerates the degradation of this ceramic CPC matrix via its acidic degradation products (i.e., lactic and glycolic acid).^10^ Previous work has shown that chemical characteristics of PLGA (i.e., acid‐terminated or end‐capped), porogen type (i.e., dense or hollow), and size all influence the degradation of CPC‐PLGA composites.^9^, ^10^, ^11^

With the use of PLGA porogens, pore formation within CPC gradually starts after ~ 2 weeks.^12^ These pores create macroporosity within the ceramic CPC matrix, which allows for fluid flow and tissue ingrowth, especially when the created pores are interconnected.^9^ Recent in vivo experimental work using a femoral condyle bone defect model in rabbits compared the degradation and bone formation of CPC‐PLGA with the dental predicate device Bio‐Oss®, which is a granulate material consisting of devitalized bovine bone.^13^ Although CPC‐PLGA demonstrated superiority regarding material degradation and bone formation after a prolonged implantation period of 6 months, initial bone formation was delayed. Earlier experimental work using a rat calvarial bone augmentation model, in which instantaneous porosity was created via a foaming technique during CPC setting and delayed porosity was created via inclusion of PLGA porogens, already showed that instantaneous porosity in CPC is favorable for initiating bone formation.^14^ These data indicate that further improvement of CPC‐porogen formulations should focus on the inclusion of porogens that rapidly dissolve or degrade to obtain macroporosity within the ceramic CPC matrix early after setting.

Alternative materials to function as a fast‐acting porogen within CPC have recently been explored.^15^, ^16^ Exploration of especially water‐soluble porogens seem appealing^15^, ^16^, ^17^, ^18^, ^19^ due to their rapid dissolution. Smith et al.^15^ showed that glucose porogens dissolve from within the ceramic CPC matrix within 3 days when submerged in phosphate‐buffered saline (PBS). Similarly, Lodoso‐Torrecilla et al.^16^ evaluated the effect of sucrose porogens, with or without PLGA porogens, in a multimodal CPC‐porogen system. Using a static in vitro degradation study set‐up, they showed rapid mass loss of CPC‐sucrose formulations. After 1 day of incubation in PBS at 37°C, this mass loss was already equivalent to the initial mass of sucrose within the CPC‐sucrose formulations, suggesting complete sucrose dissolution. Further, the CPC‐porogen formulations containing PLGA porogens showed a continuous gradual mass loss with the concomitant increase in porosity until week 6 of incubation. Yet, they observed that sucrose porogens already partially dissolved during the setting reaction, leading to pores with a considerably smaller diameter than the original sucrose particle size (i.e., size decreased from ~400 to ~130 μm). Overall, the addition of sucrose to CPC and CPC‐PLGA composites generated a CPC composite comprising the multimodal porogen platforms with accelerated pore formation and enhanced degradability, retaining injectable and cohesive properties.

We here evaluated the incorporation of multimodal porogen platforms (i.e., PLGA, sucrose, or a combination thereof) in CPC in terms of mechanical properties, dynamic in vitro degradation, and in vivo performance in a rat femoral defect model. We hypothesized that addition of sucrose porogens would enhance early CPC degradation due to fast porosity creation within the ceramic CPC matrix in contrast to delayed hydrolytic PLGA porogen degradation. As such, it was hypothesized that the addition of sucrose as a porogen to CPC would enhance CPC degradation and bone regeneration.

## MATERIALS AND METHODS

### Materials

CPC consisted of 100% milled, pure α‐TCP powder with a mean particle size of ~4.0 μm (CAM Bioceramics B.V., Leiden, The Netherlands). PLGA powder had a mean particle size of ~60 μm, a molecular weight of 17 kDa and was acid‐terminated and contained both a lactic and glycolic weight percentage of 50 (manufactured at Corbion Purac® B.V., Gorinchem, the Netherlands) (Supporting Information Fig. S1A). Sucrose had a mean particle size of ~400 μm (Merck, Darmstadt, Germany) (Supporting Information Fig. S1B). An 8 wt/vol% sodium dihydrogen phosphate dihydrate (NaH_2_PO_4_·2H_2_O) aqueous solution was used as liquid component to create the cement paste and was made by dissolving 8.0 g of NaH_2_PO_4_·2H_2_O (Merck) in 100 mL Milli‐Q water.

### Preparation of samples for characterization and dynamic degradation in vitro

CPC formulations were prepared according to the protocol described by Lodoso‐Torrecilla et al.^16^ Briefly, porogens (i.e., sucrose and/or PLGA particles) were added to α‐TCP powder in various ratios as shown in Supporting Information Table S1. For formulations containing PLGA, the ratio of CPC:PLGA remained constant at a 60:40 wt%. The addition of sucrose remained constant at 20 wt% of the total weight. This is the maximum weight percentage that can be added to CPC, retaining the handling properties required for clinical applications.^16^ An 8 wt/vol% aqueous solution of NaH_2_PO_4_ at different liquid‐to‐powder ratios (LPR) was used. To prevent premature sucrose dissolution, the aqueous solution had a temperature of 4°C. The liquid phase was added to the powder phase and mixed vigorously for approximately 20 s. The resulting paste was inserted into a mold made of polytetrafluorethylene (PTFE) containing cylindrical holes, allowing setting of the cement samples. For characterization, samples were directly analyzed or incubated in 10 mL phosphate‐buffered saline (PBS, pH = 7.4, Gibco®, Thermo Scientific, Waltham, MA) at 37°C and 120 rpm for 7 days to allow for phase transformation. Elimination of residual liquid was accomplished by freeze‐drying of the samples. Before performing the dynamic degradation tests, samples were freeze‐dried.

### Sample characterization

The morphology of the CPCs before and after 1 week of incubation was visualized by scanning electron microscopy (SEM, Zeiss Sigma 300), using a golden sputter coating for the cement samples. Imaging was performed under high vacuum at an accelerating voltage of 3 kV. The compressive strength of all CPC formulations was tested via placement in a testing bench (Ametek, Lloyd instruments, Bognor Regis, UK) and compression at a cross‐head speed of 0.5 mm/min. The compressive strength was measured before and after 7 days of incubation (*n* = 10). After mechanical testing, samples were ground to powder and analyzed by powder X‐ray diffraction (XRD; X'Pert^3^ Powder, PANalytical, Almelo, The Netherlands) to determine the crystal phase of the cement composites from 20° to 60° 2θ with a step size of 0.02° 2θ and a counting time of 1 s. An X‐ray diffractogram of α‐TCP powder was used as control.

### Set‐up for testing the dynamic degradation in vitro

For the degradation studies, a dynamic flow system was designed as described previously,^20^ with modifications. The dynamic flow system consisted of a circuit formed by several flow chambers with a cylindrical exterior (length = 62 mm, diameter = 30 mm) and a cylinder‐shaped interior (length = 62 mm, diameter = 11 mm) made of polymethylmethacrylate (PMMA). Four parallel circuits were used, one for each CPC formulation studied. Each circuit consisted of two flow chambers, each one containing two CPC samples, which were previously freeze‐dried. The flow chambers were connected using silicon tubes (L/S® Precision Pump Tubing, Masterflex®, Metrohm Applikon B.V., The Netherlands). For each group, an Erlenmeyer flask filled with 300 mL of PBS at 37°C was used as medium reservoir. A peristaltic pump (EV045, Verderflex, Verder Liquids B.V., The Netherlands) was connected to the flow chambers and the Erlenmeyer flask, which allowed PBS to flow through the samples at a rate of 1 mL/min. The dynamic flow systems were set for 1, 2, 4, 6, and 8 weeks, and after each time point the samples were taken out for further analysis and the flow system was cleaned up and started for a new time point.

### Analysis of samples subjected to dynamic degradation in vitro

To quantify PLGA degradation, the pH of the PBS buffer was analyzed directly after removal of the samples, using an electrode (Orion, Sigma Aldrich) (*n* = 1). To quantify the mass loss of the CPC samples (*n* = 4) as a function of time, sample weight was measured before and after incubation in PBS (i.e., after free‐drying of the samples) and the remaining material was calculated using Eq. (1).

(1) $$\%\;\text{Remaining material}=100-\frac{m_{i}-m_{n}}{m_{i}}*100\%$$

m_n_ represents sample mass at t = n (g) and m_i_ represents sample mass at t = 0 (g).

At each time point, calcium released into the PBS was measured using the orthochresolphtalein complexone (OCPC) method (Sigma‐Aldrich Chemie B.V., Zwijndrecht, the Netherlands) (*n* = 1).

### Preparation of samples for the animal study

An 8 w/v% aqueous solution of NaH_2_PO_4_ of 4°C was mixed by spatula with one of the various combinations of α‐TCP, sucrose and/or PLGA (Supporting Information Table S1) and inserted in a polytetrafluorethylene (PFTE) mold to allow for hardening. The samples for in vivo implantation measured 2.5 mm in diameter and 5 mm in height. All samples were sterilized by gamma irradiation at a minimum dose of 25 kGy (Synergy Health Ede, B.V., Ede, The Netherlands).

### Animal model and surgical procedure

Thirty‐two skeletally mature, male, Wistar rats with a weight ranging from 200 to 250 g were used as experimental animals (*n* = 8 per experimental group, per time‐point). The animals were housed at the Central Animal Laboratory, (Radboudumc, Nijmegen, The Netherlands) with respect to the national guidelines for the care and use of laboratory animals, following the guidelines of the 2010/63/EU directive. The study was reviewed and approved in advance by the Centrale Commissie Dierproeven (CCD; Central Commission for Animal Experiments; CCD number AVD103002015227). The rats underwent a one‐session surgical procedure. Surgery was performed under general inhalation anesthesia induced by and maintained with a mixture of isoflurane and oxygen through a constant volume ventilator. After induction of anesthesia, the rats were immobilized on their back. The surgical sites were shaved and disinfected with povidone‐iodine, followed by sterile dressing. A longitudinal incision was made medially from the patella and the patella was pushed aside. Subsequently, the distal femur was made visual and the periosteum was opened and bluntly dissected of the femoral bone. Bone defects were created in the longitudinal direction of the bone using a dental drilll, increasing the diameter of the bur until a defect of 2.5 mm in diameter and 5 mm in depth was reached (Supporting Information Fig. S2). Bone defects were filled with the various pre‐set cement samples (Supporting Information Fig. S2). The patella was pushed back in place and the subcutaneous tissue was closed with resorbable single sutures (Vicryl 4.0, Ethicon, NJ). The skin was closed with staples. Materials were allocated over the 64 defects by means of randomization with respect to the fact that the right en left femur would contain different experimental materials. Carprofen (Rimadyl®, Zoetis B.V., Capelle aan de IJssel, The Netherlands;) was administered prior to surgery and for 3 days post surgery to reduce postoperative pain and decrease inflammatory response. Implantation periods were 2 and 8 weeks. The animals were sacrificed by an overdose of CO_2_. After euthanasia, femora were retrieved, excess soft tissue was removed, and excess bone was cut off with a diamond blade saw.

### Histological procedures

Fixation of specimens was carried out in a 4% phosphate‐buffered formaldehyde solution (pH 7.2), after which the specimens were dehydrated in graded series of ethanol concentrations (70–100%) and embedded in PMMA. After polymerization, thin sections of 10 μm were prepared in a cross‐sectional direction perpendicular on the longitudinal direction of the bone defect using a saw microtome with a diamond blade (Leica SP 1600, Leica Biosystems Nussloch GmbH, Nussloch, Germany). The sections were stained with methylene blue and basic fuchsine.

### Histological and histomorphometric analysis

Histological evaluation of three sections of each sample (*n* = 3) was performed using a light microscope (Leica Microsystems AG, Wetzlar, Germany). Histomorphometric analysis of the bone sections was performed by evaluating the region of interest (ROI) of these sections. To this end, a circle of 2.45 mm in diameter was superimposed over the histological image and centered in the middle of the filled bone defect (Supporting Information Fig. S3). In samples where CPC had undergone degradation, bone morphology was assessed in order to align the ROI to the border of the defect site (Supporting Information Fig. S3c). The relative area of material remnants and bone tissue (based on staining and morphology) was quantified using image analysis software (ImageJ2, National Institutes of Health, Bethesda, MD). Material degradation was expressed as the percentage of material remnants in the region of interest (ROI).

### Statistical analysis

Data are presented as mean ± standard deviation (SD) for all experimental set‐ups (i.e., in vitro and in vivo). For all in vitro experiments, sample sizes as described in this experimental section were used for statistical analyses. For the in vivo experiment, eight samples of each experimental group (*n* = 8) were used for statistical analyses after 2 weeks of implantation. After 8 weeks, sample size varied per experimental group (CPC [*n* = 7]; CPC‐sucrose [*n* = 7]; CPC‐PLGA [*n* = 6]; CPC‐PLGA‐sucrose [*n* = 8]) (Supporting Information Table S2). A Kruskal‐Wallis test with a Dunn's multiple comparison post‐hoc test was used to identify significant differences between CPC and CPC‐sucrose and between CPC‐PLGA and CPC‐PLGA‐sucrose in all experimental set‐ups in vitro and in vivo. A Mann–Whitney test was used to identify significant differences in vivo from 2 to 8 weeks for all groups individually. Results were considered significant at *p* < 0.05. Statistical analyses were performed with GraphPad Prism 7 (GraphPad Software Inc., San Diego, CA).

## RESULTS

### Characterization of CPC formulations

SEM images (Supporting Information Fig. S4) were taken before and after 1 week of incubation. The images revealed that the CPC matrix and PLGA particles remained intact during the first week of incubation. Sucrose particles, on the other hand, were completely dissolved.

**Figure 1:**
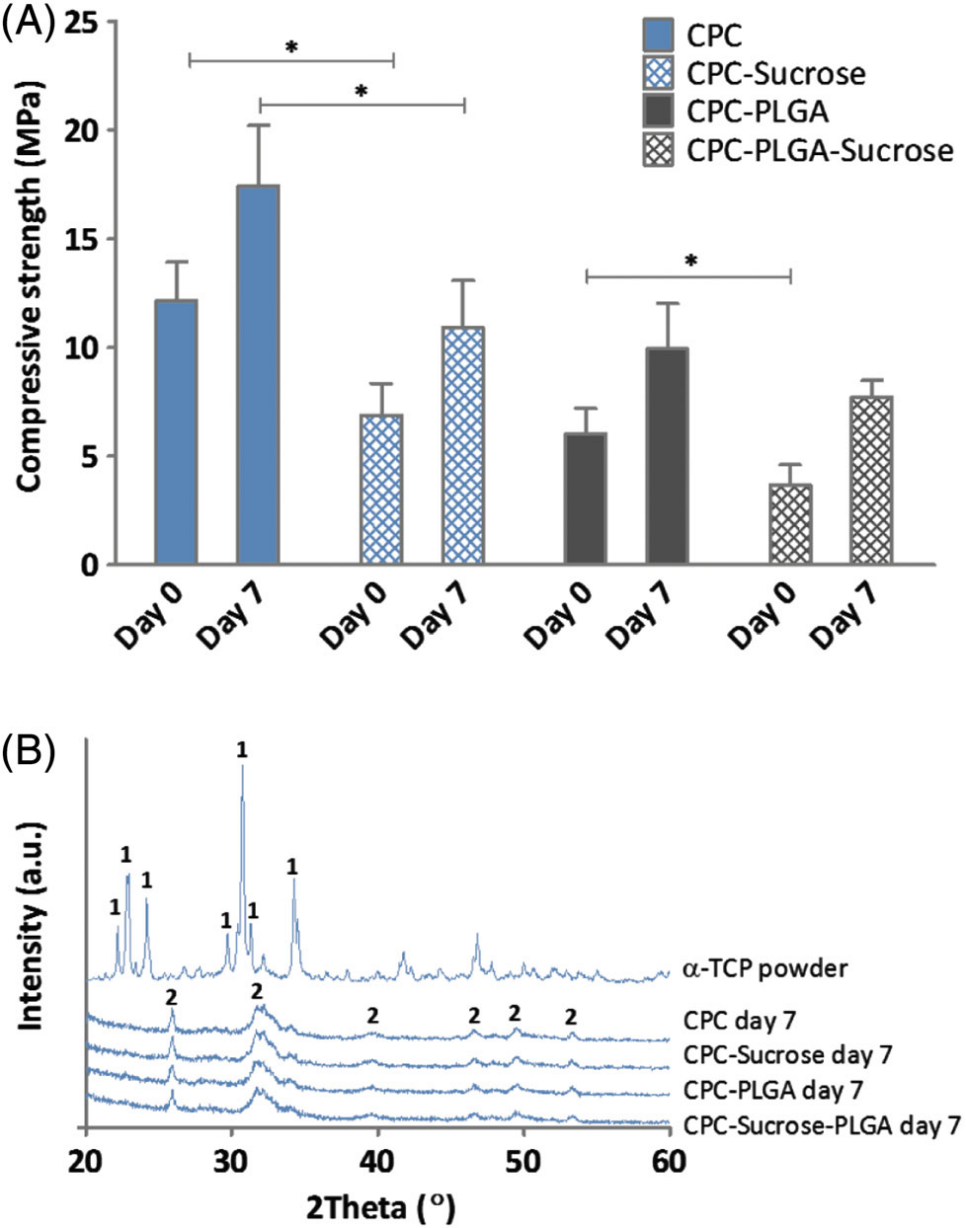
(A) Characterization of CPC formulations. Compressive strength (MPa) of the different CPC‐porogen formulations upon setting (*n* = 10) (*p < 0.05; error bars represent standard deviation (SD); Kruskal‐Wallis test with a Dunn's multiple comparison post‐hoc test) and (B) XRD patterns of α‐TCP powder (control) and the different CPC‐porogen formulations after 7 days of incubation, showing transition of α‐TCP (1) to hydroxyapatite (HA; 2) upon immersion in aqueous solution for all formulations.

The mechanical properties of the different CPC formulations were studied by means of their compressive strength (Fig. 1A). For non‐incubated samples, it was observed that pure CPC had a compressive strength of 12.2 ± 1.8 MPa, while addition of sucrose porogens to CPC significantly decreased the compressive strength to 6.9 ± 1.5 MPa (*p* < 0.05). Non‐incubated CPC‐PLGA had a compressive strength of 6.0 ± 1.2 MPa. Addition of sucrose to CPC‐PLGA significantly decreased the compressive strength to 3.7 ± 0.9 MPa (*p* < 0.05).

After 7 days of incubation in PBS, CPC had a compressive strength of 17.4 ± 2.8 MPa. Compared to CPC, addition of sucrose significantly decreased the compressive strength to 10.9 ± 2.2 MPa (*p* < 0.05). CPC‐PLGA and CPC‐PLGA‐sucrose showed similar values for compressive strength (9.9 ± 2.1 MPa and 7.7 ± 0.8 MPa, respectively; *p* > 0.05). All the groups presented a significantly higher compressive strength after 7 days of incubation compared to before incubation (*p* ≤ 0.0001 for all formulations).

Figure 1B shows the powder XRD‐patterns of CPC preincubation and all CPC‐porogen formulations after 7 days of incubation. XRD of CPC preincubation shows the main diffraction peaks of α‐TCP.^21^ XRD of the different CPC‐porogen formulations after 7 days of incubation shows similar patterns for all formulations, with the main diffraction peaks of hydroxyapatite (HA),^22^ indicating the transition of α‐TCP to HA after 7 days of incubation for all formulations.

### Dynamic degradation in vitro

**Figure 2:**
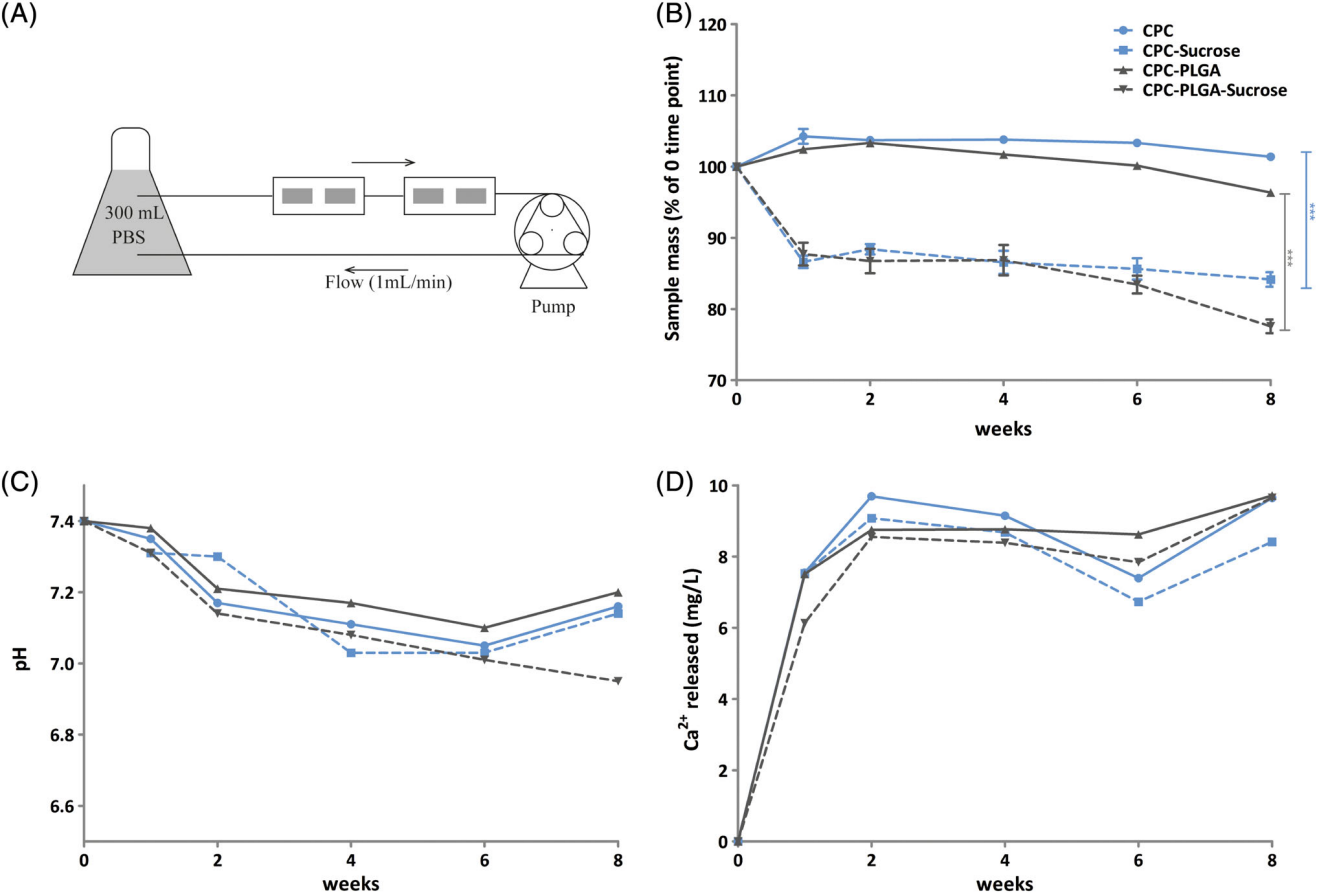
(A) Schematic representation of the dynamic flow system used for in vitro degradation studies. For each CPC formulation, four samples were used in serial configuration (two per flow chamber) and a single peristaltic pump. (B) Mass loss of CPC formulations over incubation time (*n* = 4) (*p < 0.05; error bars represent standard deviation (SD); Kruskal‐Wallis test with a Dunn's multiple comparison post‐hoc test). (C) pH of the incubation medium (PBS) over incubation time (*n* = 1). (D) Ca2+ concentration (mg/L) in the incubation medium (PBS) over incubation time (*n* = 1) (error bars represent standard deviation [SD]).

In vitro degradation was studied using a dynamic degradation set up (Fig. 2A). Degradation was evaluated using mass loss and pH measurements during 8 weeks of incubation (Fig. 2B and C, respectively). After 1 week of incubation, CPC and CPC‐PLGA showed a slight increase in mass (increase of 4.2 ± 1.0% and 2.6 ± 0.1%, respectively). CPC‐sucrose showed a significantly higher mass loss compared to CPC (mass loss of 13.4 ± 0.9% for CPC‐sucrose; *p* < 0.001). CPC‐PLGA‐sucrose showed similar values compared to CPC‐sucrose (mass loss of 12.3 ± 1.6% for CPC‐PLGA‐sucrose; *p* > 0.05).

After 8 weeks of incubation, CPC‐sucrose (mass loss of 17.5 ± 3.4%) showed a significantly higher mass loss compared to CPC (increase in mass of 1.4 ± 0.1%; *p* < 0.001). PLGA porogens induced mass loss after 8 weeks of incubation, both in CPC‐PLGA and CPC‐PLGA‐sucrose composites (mass loss of 3.6 ± 0.4% and 22.4 ± 1.0%, respectively). PLGA degradation induced a similar mass loss of ~5% in the PLGA‐containing groups when compared to their PLGA‐free counterparts.

Regarding pH measurements (Fig. 2C), CPC and different CPC‐porogen formulations showed a similar profile, with a continuous decrease in pH from 7.4 to ~7 after 6 weeks of incubation in PBS. Figure 2D shows the calcium concentration in mg/L for CPC and the different CPC‐porogen formulations during incubation in PBS. All formulations presented a similar release pattern, with an initial calcium release at week 1 of incubation of ~6–7 mg/L; which represents the ~3–4% of total calcium existent in the samples before incubation. The calcium concentration remained relatively stable over incubation time.

### General evaluation of surgical procedures and animals

The surgical procedure was uneventful for all animals and mobility was regained within 1 day after surgery without the occurrence of wound complication. At each time point (i.e., 2 and 8 weeks), 18 rats were sacrificed, from which in total 72 specimens were retrieved. Of these, four specimens were excluded for histological analyses due to inferior staining quality. An overview of the number of implants that were placed, retrieved, and analyzed per experimental group is presented in Supporting Information Table S2.

### Descriptive histology 2 week implantation period

**Figure 3:**
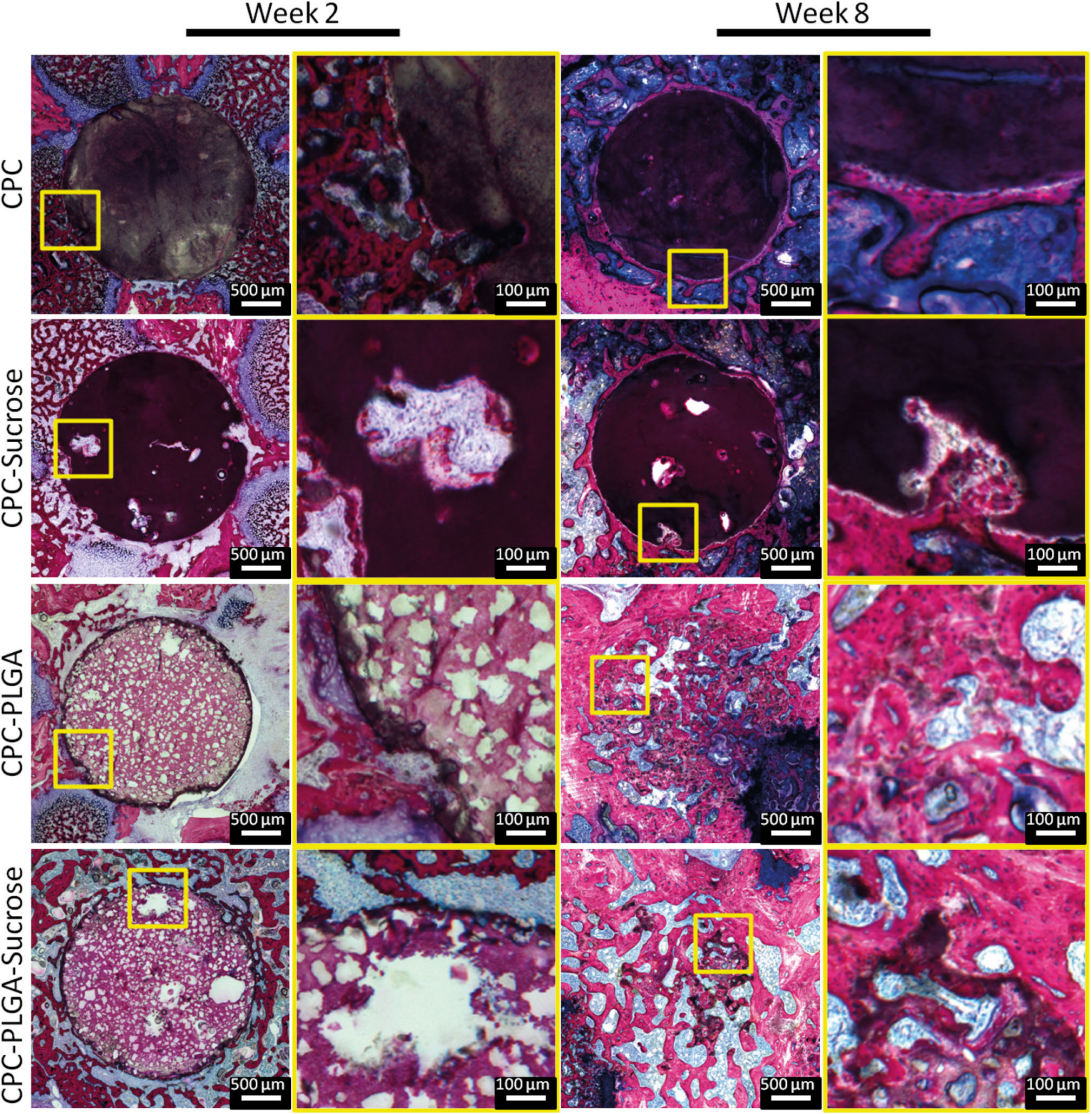
Histological overview and magnifications of CPC formulations implanted in a rat femoral bone defect at two (left panels) and 8 weeks (right panels). Sucrose porogen and PLGA porogen or pores resulting from sucrose porogen dissolution (large) or PLGA porogen degradation (small) can be discriminated based on size differences. Large pores resulting from sucrose porogen dissolution are not homogeneously distributed. At 2 weeks, limited tissue infiltration in peripheral pores resulting from sucrose porogen dissolution is apparent. At 8 weeks, significant degradation of PLGA‐containing CPC formulations can be observed. Pure CPC shows hardly any degradation over the entire implantation period.

The left panels of Figure 3 present a histological overview of all CPC‐formulations after 2 weeks of implantation. Implant integrity was maintained for the implants of all experimental CPC formulations. For CPC, no signs of material degradation were observed. CPC‐sucrose showed large pores, surrounded by CPC with a dense appearance. For CPC‐sucrose, a thin layer of bone was observed in pores located at the implant periphery. CPC‐PLGA and CPC‐PLGA‐sucrose had a porous appearance. Presence of PLGA porogen could not be evidently distinct from presence of pores as a result of PLGA porogen degradation. For CPC‐PLGA‐sucrose, large pores were visible within the ceramic CPC matrix. These pores were not homogeneously distributed. No fibrous tissue was observed within or in the vicinity of the experimental implants.

### Descriptive histology 8 week implantation period

The right panels of Figure 3 present a histological overview of all CPC‐formulations after 8 weeks of implantation. CPC and CPC‐sucrose showed an irregular border as a result of marginal peripheral material degradation. New bone formation was observed at the periphery of the experimental implants, while the center of the defect still contained ceramic CPC matrix. For CPC‐sucrose, newly formed bone was observed in the pores. A substantial decrease in material remnants was observed for CPC‐PLGA as well as for CPC‐PLGA‐sucrose. For these CPC‐porogen formulations, remnants were diffusely distributed throughout the defect area. New bone formation was observed in the areas of the degraded material, at the periphery of the defect, and in the center.

### Histomorphometric results concerning material degradation

**Figure 4:**
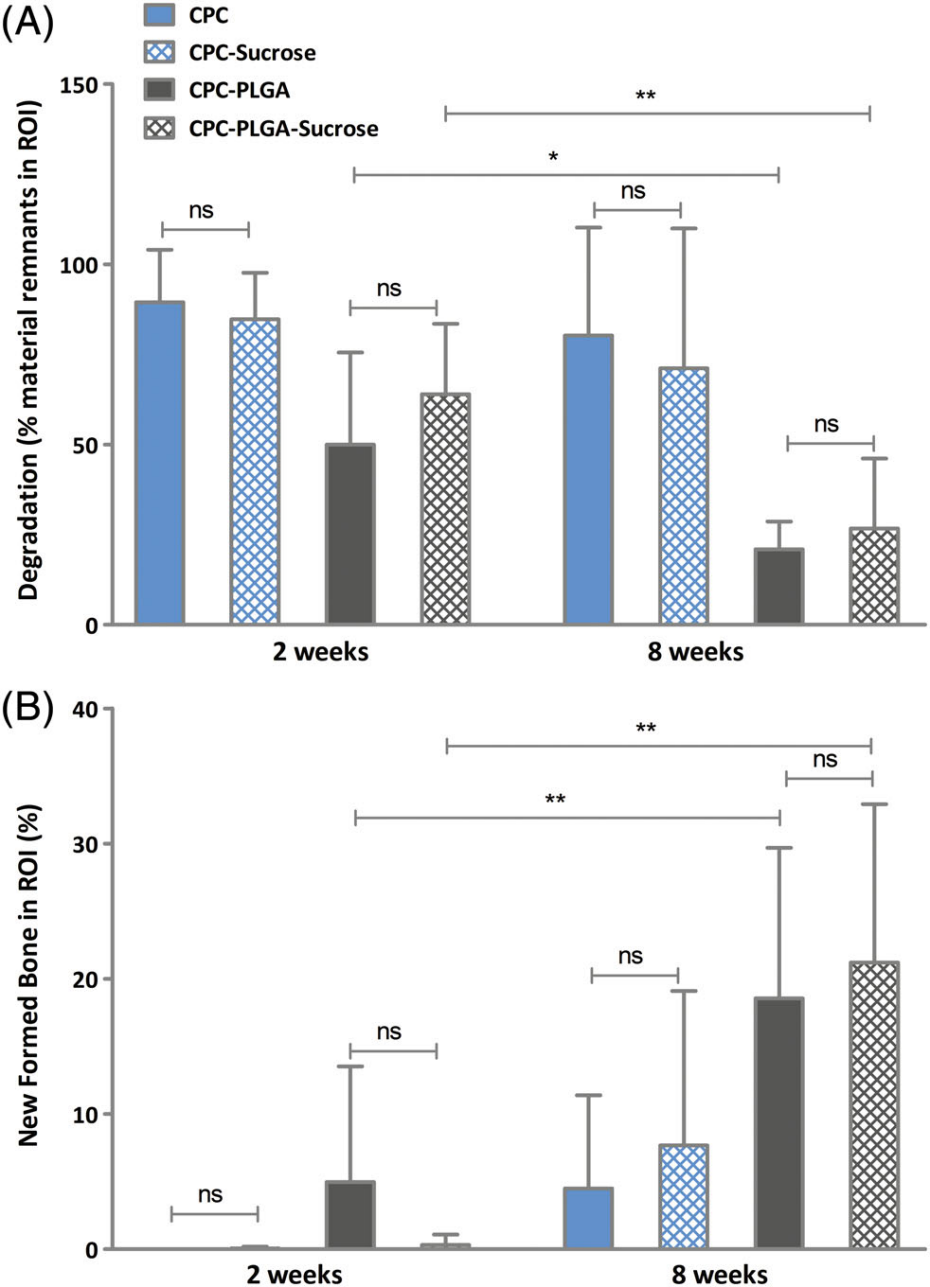
Histomorphometrical quantification of material remnants (A) and new formed bone (B) in the region of interest (ROI) for CPC‐sucrose, CPC‐PLGA, CPC‐PLGA‐sucrose and CPC after two (*n* = 8) and 8 weeks (*n* = 7, *n* = 6, *n* = 8, and *n* = 7, respectively) of implantation in a rat femoral bone defect (error bars represent standard deviation [SD]). (*p < 0.05; error bars represent standard deviation [SD]; Kruskal‐Wallis test with a Dunn's multiple comparison post‐hoc test).

Histomorphometric results concerning material degradation are presented in Figure 4A. In 2 weeks, CPC (89.5 ± 14.6%) showed similar material remnant values compared to CPC‐sucrose (84.8 ± 12.9%; *p* > 0.05). CPC‐PLGA (50.0 ± 25.5%) showed similar material remnant values compared to CPC‐PLGA‐sucrose (64.0 ± 19.5%; *p* > 0.05).

In 8 weeks, CPC (80.3 ± 29.9%) showed similar material remnant values compared to CPC‐sucrose (71.1 ± 38.8%; *p* > 0.05). CPC‐PLGA (20.9 ± 7.7%) showed similar material remnant values compared to CPC‐PLGA‐sucrose (26.8 ± 19.4%; *p* > 0.05). From 2 to 8 weeks, CPC‐PLGA and CPC‐PLGA‐sucrose showed a significant decrease in material remnant values (*p* = 0.03 and *p* = 0.007, respectively), while for CPC and CPC‐sucrose material remnant values were similar at 2 and 8 weeks (*p* > 0.05).

### Histomorphometric results concerning bone formation

Histomorphometric results concerning new bone formation are presented in Figure 4B. In 2 weeks, CPC (0 ± 0%) showed a similar amount of new formed bone compared to CPC‐sucrose (0.1 ± 0.1%; *p* > 0.05). CPC‐PLGA (5.0 ± 8.6%) showed a similar amount of newly formed bone compared to CPC‐PLGA‐sucrose (0.3 ± 0.8% *p* > 0.05).

In 8 weeks, CPC (4.5 ± 6.9%) showed a similar amount of new formed bone compared to CPC‐sucrose (7.7 ± 11.4%; *p* > 0.05). CPC‐PLGA (18.6 ± 11.1%) showed a similar amount of new formed bone compared to CPC‐PLGA‐sucrose (21.2 ± 11.7%; *p* > 0.05). From 2 to 8 weeks, CPC‐PLGA and CPC‐PLGA‐sucrose showed a significant increase in new bone formation (*p* = 0.01 and *p* < 0.002, respectively), while for CPC and CPC‐sucrose new bone formation was similar at 2 and 8 weeks (*p* > 0.05).

## DISCUSSION

We here evaluated multimodal porogen platforms for incorporation within CPC to introduce porosity within the ceramic CPC matrix and accelerate material degradation and bone formation. In vitro, we assessed the mechanical properties and dynamic degradation characteristics of the different CPC‐porogen formulations. Furthermore, we performed an in vivo study to evaluate the biological performance of the different CPC‐porogen formulations in terms of degradation and concomitant bone formation. We hypothesized that the inclusion of sucrose porogens in CPC would accelerate early stage material degradation and bone regeneration due to fast porogen dissolution. Additionally, we hypothesized that late stage degradation and bone formation would be enhanced by (delayed) hydrolytic degradation of PLGA porogens and the acidifying effects of the degradation products thereof on the ceramic CPC matrix. The experimental data showed that inclusion of sucrose porogens decreased the initial compressive strength of the CPC and induced an early in vitro mass loss equal to the quantity of sucrose. Further, the addition of sucrose did not alter the crystallographic profile. Upon application as a bone substitute material in a rat femoral bone defect model, no additional effects were observed from the incorporation of sucrose porogens in CPC or CPC‐PLGA.

Compressive strength measurements for the different CPC formulations upon setting showed that the inclusion of sucrose porogens significantly decreased the initial compressive strength (i.e., before incubation in PBS), while the compressive strength of CPC‐PLGA‐sucrose was similar to that of CPC‐PLGA after 7 days of incubation in PBS. In general, it has been widely reported that the inclusion of porogens in CPCs, and the subsequent creation of a macroporous ceramic structure, reduces the mechanical properties of CPCs.^11^, ^23^, ^24^ Zhang et al. evaluated around 20 different studies and observed a negative correlation between the porosity percentage in CPCs and their compressive strength.^24^ On the other hand, all CPC formulations showed significantly higher compressive strength after incubation in PBS for 7 days due to the phase transformation from α‐TCP to hydroxyapatite, as confirmed by XRD results.^25^ Furthermore, it is important to take into account that in vivo, sucrose will dissolve within the first day and, subsequently, the mechanical properties of the CPC might become temporarily negatively affected until the phase transformation occurs.^26^

In order to test new pore‐forming additives for enhancement of CPC degradation, in vitro degradation studies are commonly performed. While most studies analyze the in vitro degradation using a static set up (where the samples are introduced in a soaking medium without any mechanical stirring or fluid flow),^10^, ^27^, ^28^ others use a dynamic set up (where the samples are incubated under a dynamic flow of medium).^20^, ^29^, ^30^ It has been speculated that dynamic set‐ups are more biomimetic than static set‐ups.^20^, ^29^ For CPC‐PLGA constructs, most in vitro studies performed to data have used a static set up with sample incubation in a small amount of soaking medium, which magnifies the acidification effect related to PLGA degradation on the ceramic matrix of the CPC.^10^, ^16^, ^31^ On the other hand, dynamic degradation set‐ups commonly use higher amounts of soaking medium, which, together with the continuous fluid flow, act as a buffer and the PLGA‐related acidification only has a minor local effect, similar to the in vivo situation.^20^, ^29^ In view of this, we used a dynamic set up to study in vitro degradation of the different CPC formulations during 8 weeks. As hypothesized, sucrose dissolution occurred at an early stage while PLGA degradation did not occur until approximately 6 to 8 weeks of incubation. In previous research,^16^ using a static degradation set‐up the dissolution of sucrose also occurred within the first week of incubation, but PLGA degradation started at an earlier time point (i.e., after approximately 2 weeks of incubation). This difference between dynamic and static set‐ups was also observed by An et al. and Agrawal et al.,^20^, ^29^ which is likely due to the accelerating effect of a lower pH on PLGA degradation. This higher acidification in static set‐ups is related to both the difference in soaking medium amounts used (up to 10 mL in static vs. up to 300 mL in dynamic) and the buffering effect of the fluid flow created in the dynamic set up.

Based on the results of the in vitro studies, two different implantation periods of 2 and 8 weeks were selected. Similar to previous studies, 2 weeks was selected as an early stage implantation time point to observe the effect of sucrose porogen dissolution on pore formation, material degradation, and bone formation.^32^, ^33^ In contrast, effects of PLGA degradation on these aspects were expected after a prolonged implantation (e.g., 8 weeks).^19^, ^32^, ^33^ Indeed, CPC implants containing sucrose porogens showed the presence of large pores within the ceramic CPC matrix after 2 weeks of implantation. This observation matches the in vitro dynamic dissolution study, where it was observed that after 1 week of incubation the sucrose was fully dissolved. However, while in vitro results showed that PLGA starts degrading after 6–8 weeks of incubation, in vivo results showed otherwise. Here, PLGA started degrading after 2 weeks of implantation, similar to what was observed by Renno et al.,^33^ and after 8 weeks only ~20% of the material was remaining. On the other hand, static in vitro data previously revealed that PLGA dissolution started after 2 weeks of incubation, which more closely correlates with the in vivo situation.^16^

A limitation of this study is not being able to fully distinct between non‐degraded PLGA porogen and pores as a result of PLGA porogen degradation. We quantified the relative area of material remnants (i.e., the amount of CPC) based on staining and morphology. According to previous studies,^9^, ^32^, ^33^ the porous appearance in the formulations containing PLGA at 2 weeks, should correspond to non‐degraded PLGA porogen. As a result, measurements of material remnants in the PLGA‐containing formulations might be underestimated.

While sucrose dissolution occurred at an early stage in the dynamic degradation set up in vitro, sucrose porogens did not improve the biological performance of CPC or CPC‐PLGA in terms of early material degradation and concomitant bone formation. Regarding this discrepancy, the influence of particle size and manner of disappearance of both porogen platforms should be considered. In this study, PLGA particles had a mean particle size of ~60 μm and sucrose particles had a mean particle size of ~400 μm. PLGA particles disappear upon hydrolytic degradation, creating an acidic environment which accelerates the degradation of the CPC matrix,^8^, ^34^ increasing pore size compared to initial particle size. Sucrose particles on the other hand, disappear upon dissolution. Previous work^16^ showed that sucrose particles suffer partial dissolution during the process of cement setting, starting when the liquid component is added to the powder component. The consequences of premature particle dissolution are a decreased pore size compared to initial particle size and a decreased weight percentage of sucrose available to form macropores after setting. It was previously described that to generate an interconnected pore structure, the volume fraction of porogens should exceed 40%.^12^, ^15^ Del Real et al.^35^ stated that CaP ceramics should have interconnective pores with a diameter larger than 100 μm to allow for active resorption throughout the CaP matrix. A clear effect of the loading amount and size of PLGA porogen on interconnectivity within CPC was observed by Lopez‐Heredia et al.^11^ using interconnectivity μ‐CT analysis. They observed that an increased particle size might reduce the loading amount without a change in interconnectivity, although general porosity of CPC would be increased by the use of smaller particles, generating faster resorption. Previous in vivo studies showed the influence of particle size of PLGA porogen on the biological performance of CPC. Liao et al.^36^ observed significant effects on the biological performance of CPC‐PLGA by altering the size of hollow PLGA microspheres. CPC with small, hollow microspheres showed a twofold higher bone formation compared to large, hollow microspheres after 12 weeks of implantation. Hoekstra et al.^37^ found no effect of dense PLGA microsphere size on material degradation and bone formation after 12 weeks of implantation. For CPC containing the sucrose porogen platform, an improvement in pore interconnectivity would enable fluid flow throughout the material and hence active material degradation. In view of this, smaller porogens would be preferable for the sucrose porogen platform, because of the increase in interconnectivity when homogeneously distributed. Ideally, dissolution of sucrose porogens should be delayed (e.g., by cooling the liquid component used for CPC preparation) until initial CPC setting has occurred.

## CONCLUSION

Inclusion of the sucrose porogen platform in CPC decreased the compressive strength of CPC and induced an early in vitro mass loss proportional to the amount of sucrose. Inclusion of the sucrose porogen platform in CPC‐PLGA did not decrease the compressive strength of CPC‐PLGA after 7 days of incubation and did not affect in vitro mass loss. Evaluation of the biological performance in a rat femoral bone defect showed that inclusion of the sucrose porogen platform in CPC or CPC‐PLGA had no additional effects in terms of early degradation or additional bone formation.

## CONFLICT OF INTEREST

The authors declare no potential conflict of interest. No benefit of any kind will be received either directly or indirectly by the authors.

## Supporting information


**Table S1** Material components per experimental group: wt% α‐TCP, PLGA and Sucrose per group.
**Table S2**. Overview of the number of orthotopic implants placed, retrieved and analyzed per group after 2 and 8 weeks.
**Figure S1**. Overview of the porogens used. A) Representative light microscopy image of sucrose particles and B) representative SEM image of PLGA particles.
**Figure S2**. Overview of the surgical procedure. From left to right: creating the defect; the created defect (Ø 2.5 mm); medial lateral view of the distal femur with the pre‐set scaffold halfway inserted into the created defect; caudal view of the distal femur with the pre‐set scaffold fully inserted into the created defect.
**Figure S3**. Histological section showing the circular region of interest (ROI) that was superimposed over histological sections for quantification of the histomorphometrical parameters material remnants and bone formation. Representative ROIs superimposed over A) an intact CPC and B) a degraded CPC. C) Alignment of the ROI to the border of the defect site when CPC was degraded.
**Figure S4**. Representative SEM images of CPC (A and E), CPC/Sucrose (B and F), CPC/PLGA (C and G) and CPC/PLGA/Sucrose (D and H) formulations before incubation (week 0; A, B, C, and D), and after 1 week incubation (E, F, G and H). White bar represents 100 μm.
