# Supplementary material for: Multimodal porogen platforms for calcium phosphate cement degradation
Source: J Biomed Mater Res A. 2019 Apr 9;107(8):1713–22. doi: 10.1002/jbm.a.36686 (PMC6618311; doi:10.1002/jbm.a.36686)
Supplement: Supplementary file 1 — Table S1 Material components per experimental group: wt% α‐TCP, PLGA and Sucrose per group. Table S2. Overview of the number of orthotopic implants placed, retrieved and analyzed per group after 2 and 8 weeks. Figure S1. Overview of the porogens used. A) Representative light microscopy image of sucrose particles and B) representative SEM image of PLGA particles. Figure S2. Overview of the surgical procedure. From left to right: creating the defect; the created defect (Ø 2.5 mm); medial lateral view of the distal femur with the pre‐set scaffold halfway inserted into the created defect; caudal view of the distal femur with the pre‐set scaffold fully inserted into the created defect. Figure S3. Histological section showing the circular region of interest (ROI) that was superimposed over histological sections for quantification of the histomorphometrical parameters material remnants and bone formation. Representative ROIs superimposed over A) an intact CPC and B) a degraded CPC. C) Alignment of the ROI to the border of the defect site when CPC was degraded. Figure S4. Representative SEM images of CPC (A and E), CPC/Sucrose (B and F), CPC/PLGA (C and G) and CPC/PLGA/Sucrose (D and H) formulations before incubation (week 0; A, B, C, and D), and after 1 week incubation (E, F, G and H). White bar represents 100 μm. [file JBM-107-1713-s001.docx]

Supporting Information

**Multimodal porogen platforms for calcium phosphate cement degradation**

Irene Lodoso-Torrecilla ^1,#^, Eline-Claire Grosfeld ^1,#^, Abe Marra ^1^, Brandon T Smith ^2^, Antonios G Mikos ^2,3^, Dietmar JO Ulrich ^4^, John A Jansen ^1^, Jeroen JJP van den Beucken ^1,*^

## 1. Supplemental Tables

Table S1. Material components per experimental group: wt.% α-TCP, PLGA and Sucrose per group.

|  | | CPC-Sucrose | | CPC-PLGA | | CPC-PLGA-Sucrose | | CPC | |
| --- | --- | --- | --- | --- | --- | --- | --- | --- | --- |
| α-TCP | 80 | | 60 | | 48 | | 100 | |  |
| PLGA | 0 | | 40 | | 32 | | 0 | |  |
| Sucrose | 20 | | 0 | | 20 | | 0 | |  |
| LPR | 0.3 | | 0.41 | | 0.23 | | 0.47 | |  |

Table S2. Overview of the number of orthotopic implants placed, retrieved and analyzed per group after 2 and 8 weeks.

| 2 weeks | | | | | 8 weeks | | | |
| --- | --- | --- | --- | --- | --- | --- | --- | --- |
|  | CPC-Sucrose | CPC-PLGA | CPC-PLGA-Sucrose | CPC | CPC-Sucrose | CPC-PLGA | CPC-PLGA-Sucrose | CPC |
| Placed (n) | 8 | 8 | 8 | 8 | 8 | 8 | 8 | 8 |
| Retrieved (n) | 8 | 8 | 8 | 8 | 8 | 8 | 8 | 8 |
| Analyzed (n) | 8 | 8 | 8 | 8 | 7 ^a)^ | 6 ^a)^ | 8 | 7 ^a)^ |

^a)^ due to inferior quality of the sample

## 2. Supplemental Figures


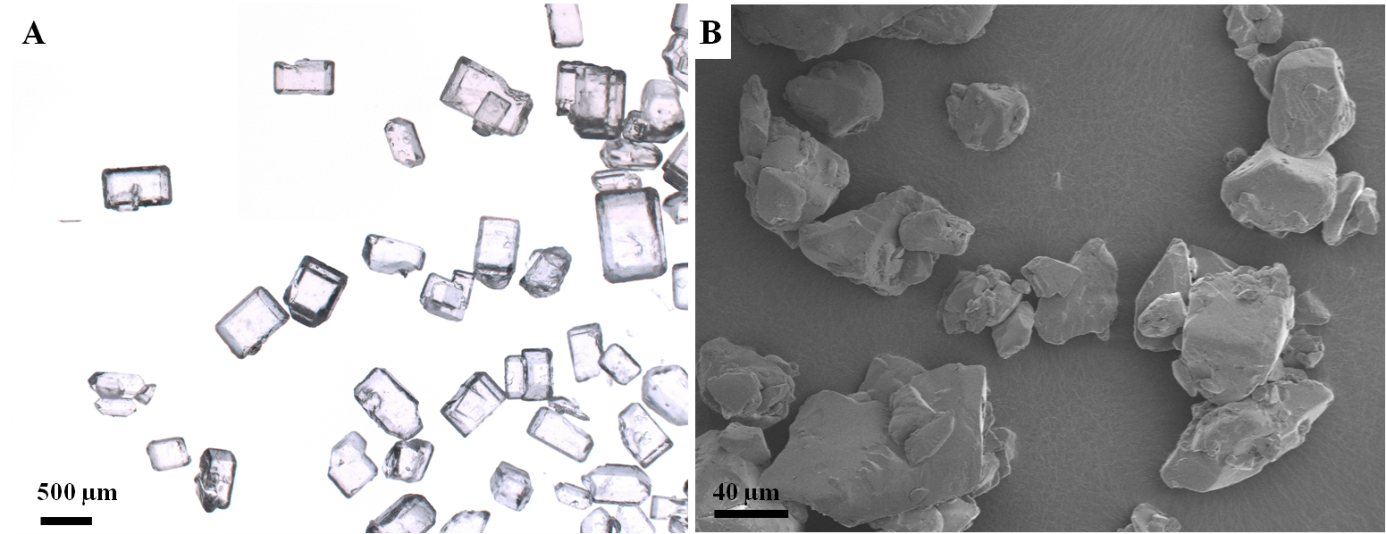


Figure S1. Overview of the porogens used. A) Representative light microscopy image of sucrose particles and B) representative SEM image of PLGA particles.


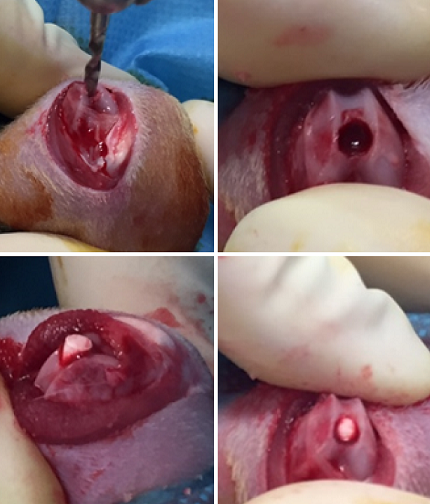


Figure S2. Overview of the surgical procedure. From left to right: creating the defect; the created defect (Ø 2.5 mm); medial lateral view of the distal femur with the pre-set scaffold halfway inserted into the created defect; caudal view of the distal femur with the pre-set scaffold fully inserted into the created defect.


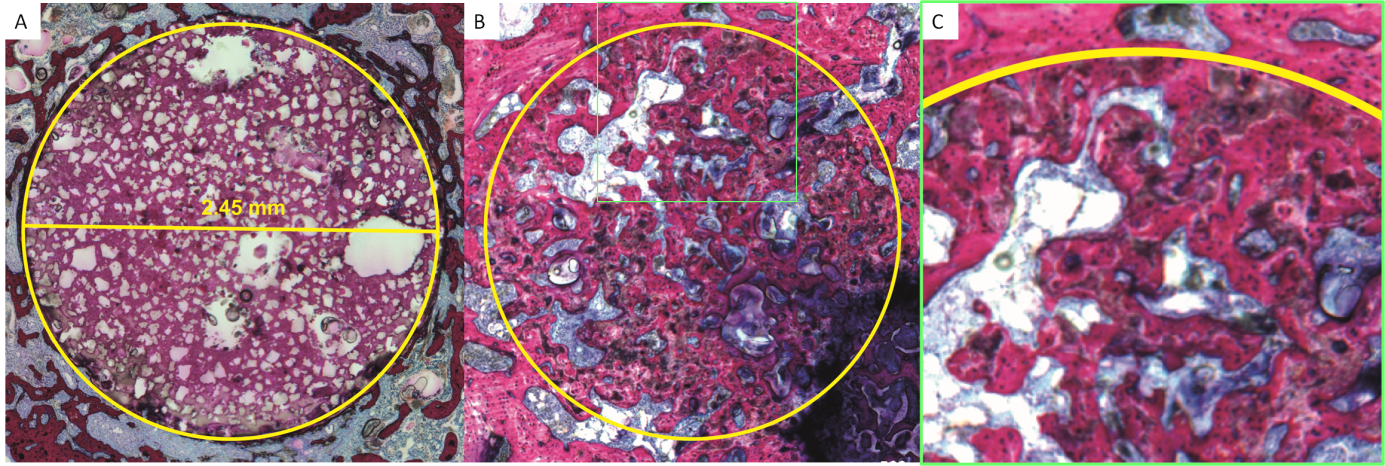


Figure S3. Histological section showing the circular region of interest (ROI) that was superimposed over histological sections for quantification of the histomorphometrical parameters material remnants and bone formation. Representative ROIs superimposed over A) an intact CPC and B) a degraded CPC. C) Alignment of the ROI to the border of the defect site when CPC was degraded.


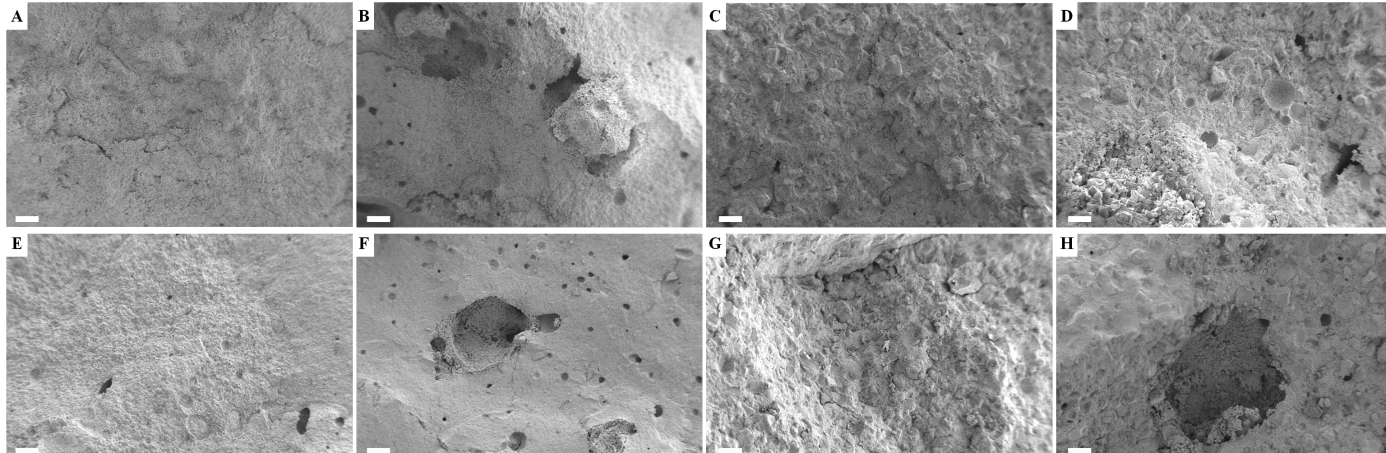


Figure S4. Representative SEM images of CPC (A and E), CPC/Sucrose (B and F), CPC/PLGA (C and G) and CPC/PLGA/Sucrose (D and H) formulations before incubation (week 0; A, B, C and D), and after 1 week incubation (E, F, G and H). White bar represents 100 µm.
